# Risk of opportunistic infections in patients with rheumatoid arthritis initiating abatacept: cumulative clinical trial data

**DOI:** 10.1186/s13075-020-02399-2

**Published:** 2021-01-11

**Authors:** Teresa A. Simon, Lixian Dong, Kevin L. Winthrop

**Affiliations:** 1 Bristol Myers Squibb, Princeton, NJ 08543 USA; 2 Current affiliation: Physicians Research Center, LLC, Toms River, NJ 08753 USA; 3 Oregon Health & Science University, Portland, 97239 OR USA

**Keywords:** Abatacept, Infection, Opportunistic infections, Herpes, Serious infection, Shingles, Rheumatoid arthritis, Tuberculosis

## Abstract

**Background:**

To evaluate incidence of opportunistic infections (OIs) in patients with rheumatoid arthritis (RA) treated with abatacept in clinical trials.

**Methods:**

This pooled analysis of 16 randomized, double-blind/open-label trials, with ≥ 1 abatacept (intravenous or subcutaneous) arm, and with/without placebo control covered cumulative (controlled short-term and open-label long-term) abatacept exposure periods. OIs were analyzed separately in controlled (abatacept and placebo individually) and cumulative periods. OIs were identified using a prespecified list; events were independently adjudicated. Unadjusted incidence rates (IRs; per 100 patient-years) with 95% confidence intervals (CIs) were calculated.

**Results:**

In cumulative periods, 7044 patients received abatacept, with a mean (standard deviation) duration of exposure of 36.9 (26.2) months (21,274 patient-years of exposure). IRs (95% CIs) of OIs were 0.17 (0.05–0.43) for abatacept and 0.56 (0.22–1.15) for placebo during the controlled periods and 0.21 (0.15–0.28) for abatacept during the cumulative periods. There was 1 case of tuberculosis in both the abatacept (IR [95% CI] 0.04 [0.00–0.24]) and placebo (IR [95% CI] 0.08 [0.00–0.44]) groups during the controlled periods; 13 verified tuberculosis cases (IR [95% CI] 0.06 [0.03–0.10]) were reported in the cumulative period. Herpes zoster was reported numerically more often with abatacept (IR 1.9 [1.4–2.5]), versus placebo (1.7 [1.1–2.6]) in the controlled periods; within the cumulative period, herpes zoster IR (95% CI) was 1.53 (1.36–1.71) for abatacept-treated patients.

**Conclusion:**

In controlled periods of the clinical trials, abatacept-treated patients had similarly low rates of OIs compared with placebo-treated patients. Overall, OI rates were similar among abatacept-treated patients in the controlled and cumulative periods and consistent with the ranges reported in the literature.

## Background

Biologic disease-modifying antirheumatic drugs (bDMARDs) are effective treatments for rheumatoid arthritis (RA). Currently available bDMARDs include those that target cytokines, such as tumor necrosis factor (TNF), interleukins, and peripheral B cells, and T cell activation. The mechanisms of action of currently available bDMARDS (targeting B cells, cytokines, T cells, and interleukins) can increase the risk of serious infections and opportunistic infections (OIs) in patients with RA. Patients with RA are already at an increased risk of infections compared with patients without RA [1–8]. As RA is a chronic disease requiring prolonged treatment, it is important for physicians to consider the long-term safety implications of different therapies in addition to their efficacy to make informed treatment decisions.

Currently, there is a growing amount of evidence available on the safety of DMARDs in patients with RA: several real-world studies have investigated the safety of different conventional synthetic (cs) DMARDs and bDMARDs, including rates of OIs [4–7].

Abatacept, a selective T cell co-stimulation modulator with a distinct mechanism of action upstream of other bDMARDs [9], is an effective and well-tolerated treatment for patients with RA [10–15]. Data from the original clinical development program of abatacept in RA includes results from 7 randomized controlled trials (RCTs). The incidence rates (IRs) of infections in these trials were consistent with the rates observed in reference cohorts of patients with RA treated with non-biologic DMARDs [16]. In an integrated analysis of 9 double-blind, placebo-controlled RCTs (7 intravenous [IV] and 2 subcutaneous [SC]) of abatacept in RA, the IRs of adverse events (AEs), including OIs, were comparable between abatacept and placebo groups [17]. Understanding the risk of OIs with abatacept was limited by the short durations of exposure within these datasets; as such, we chose to evaluate the risk of OIs using all available randomized, controlled, clinical trial data and long-term extension data from the RA abatacept development program. This analysis provides a comprehensive evaluation of OIs to supplement the previously published overall safety analysis of abatacept [17].

## Methods

### Study design

Overall, 16 abatacept clinical trials sponsored by Bristol Myers Squibb prior to June 2016 were included in this analysis. Early phase, pharmacokinetic, and country-specific studies were not considered, as they are not representative of a global RA population.

The trials included in this evaluation were either randomized, double-blind trials (*n* = 10) or open-label trials (*n* = 6; 7/10 double-blind trials with an open-label extension period), and at least one dose of abatacept (IV or SC).

The controlled period included all patients randomized in the double-blind portion of the 16 studies. The cumulative period included all patients treated with abatacept from the double-blind and open-label periods, as well as patients randomized to placebo and treated with abatacept in the open-label period. The start of the period was defined as the day of the first dose of study medication. All patients who received ≥ 1 dose of abatacept or placebo were included in the safety analysis. Patients receiving active comparator (infliximab in the ATTEST trial [*n* = 165], adalimumab in the AMPLE trial [*n* = 328] were excluded from this analysis.

Across all 16 trials, patients randomized to placebo were receiving a non-biologic DMARD. Details on the studies are summarized in Additional File 1: Supplementary Table 1.

Eligibility criteria were largely consistent across the included studies [17]. The main inclusion criteria common to all 16 trials were a patient age of ≥ 18 years, a diagnosis of RA [18], and permitted prior corticosteroid use. Relevant exclusion criteria included active tuberculosis (TB) requiring treatment within the previous 3 years, and screening for TB involved a tuberculin skin test or QuantiFERON® test in addition to a chest roentgenogram. Patients with a positive TB screening test indicative of latent TB were ineligible for the study unless active TB infection had been ruled out and treatment for latent TB with isoniazid had been initiated at least 4 weeks before administration of the study drug; such patients were required to complete 9 months of isoniazid treatment during the study.

### Study assessments

All studies were carried out and reported in accordance with the Declaration of Helsinki and International Conference on Harmonization Good Clinical Practice guidelines [19]. All patients were monitored for the occurrence of AEs, serious adverse events (SAEs), AEs leading to discontinuation of study treatment, and deaths. Events were classified using the version of the Medical Dictionary for Regulatory Activities (MedDRA) classification that was current at the time of each study. For the purpose of this research, relevant events (OIs) were reclassified using the current MedDRA version at the time of this integrated analysis (version 20.0). When counting AEs, if patients had > 1 OI, the first OI event was counted toward the overall incidence. If the same event occurred multiple times in the same patient, the most severe event was counted.

Incidence of OIs within controlled periods among abatacept- and placebo-treated patients was analyzed. Overall cumulative OI incidence included controlled and open-label periods of all studies (see Additional File 1: Supplementary Table 1). A prespecified list of OIs was used to identify the OIs (available in Additional File 1: Supplementary Appendix A: Prespecified list of OIs in clinical trials). Criteria for inclusion were based on type, location of the infection, and causative organism; more than 280 reported terms were screened as potential OIs. OI events were independently adjudicated by the author (KLW) using case definitions from the relevant consensus paper [2]. OI was reported as serious if fulfilling the regulatory criteria for an SAE (any untoward medical occurrence that at any dose of the study drug either results in death, is life-threatening, requires hospitalization or its prolongation, results in persistent or significant disability, is a congenital anomaly or an important medical event).

### Statistical analysis

Descriptive statistics as mean (standard deviation [SD]) or *n* (%) were computed for baseline demographic and clinical characteristics. Numbers of events, percentages, and unadjusted (crude) IRs (per 100 patient-years) with 95% confidence intervals (CIs) were calculated for the OIs overall and for the individual OI.

#### IR calculation for overall OIs

IR calculation of OIs included some terms or microorganisms that may not be considered opportunistic (e.g., *Pseudomonas aeruginosa*-caused pneumonia) [2]. Similarly, only disseminated or visceral herpes zoster and herpes simplex cases deemed invasive were included in the overall OI analyses. All cases of TB were included in the overall OI IR except latent TB cases.

#### IR calculation for herpes infection

The IR for herpes includes all reported cases of herpes including the cases of disseminated or visceral herpes zoster and invasive herpes simplex cases.

#### TB

Including latent TB cases in an IR calculation was deemed inappropriate by KLW (coauthor); therefore, latent TB cases were not included in the determination of the overall TB IR.

Exposure was censored at the time of the first OI event, death, discontinuation, or end of study for patients with no event, whichever was the earliest. Duration of exposure to the study drug was defined as the number of days from the start of therapy to the day of treatment cessation at the end of the controlled period (or early discontinuation) or at the end of the open-label extension period plus 56 days (or 60 days for phase II or IV trials; approximately 4 half-lives of abatacept in humans).

## Results

### Patient disposition and baseline characteristics

In total, 2653 patients received abatacept and 1485 received placebo in the controlled periods; 7044 patients received abatacept in the cumulative periods. Extent of exposure and patient baseline characteristics during the controlled periods of the clinical trials have been reported previously [17]. In the cumulative period, demographic and disease characteristics as well as concomitant medications were similar to those seen in the controlled periods for abatacept (Table 1): mean (SD) age was 51.5 (12.6) and 51.7 (12.4), respectively; 80.6% and 79.1% of patients were female. Mean (SD) duration of exposure to abatacept in the controlled and cumulative periods, respectively, was 10.8 (3.3) and 36.9 (26.2) months with 2356.6 and 21,274 total patient-years of exposure.

**Table 1:** Baseline demographics and characteristics of patients from the double-blind, placebo-controlled and cumulative periods

	Double-blind, placebo*-controlled period		Cumulative period
	Abatacept (*N* = 2653)	Placebo (*N* = 1485)	Abatacept (*N* = 7044)
**Patient demographics**			
Age, years	51.7 (12.4)	51.4 (12.3)	51.5 (12.6)
Weight, kg	73.5 (18.8) *n = 2650*	73.8 (18.6) *n = 1484*	74.4 (19.0) *n = 7036*
Female, *n* (%)	2099 (79.1)	1184 (79.7)	5675 (80.6)
White, *n* (%)	2283 (86.1)	1285 (86.5)	5940 (84.3)
Durations of exposure, months	10.8 (3.3)	10.3 (3.5)	36.9 (26.2)
**Disease characteristics**			
Disease duration, years	8.1 (8.5) *n = 2577*	7.5 (8.5) *n = 1439*	8.1 (8.6) *n* *= 6933*
hsCRP, mg/L	26.3 (29.8) *n = 2567*	27.2 (33.5) *n = 1435*	24.8 (29.8) *n = 6936*
Tender joint count (28)	29.8 (13.9) *n = 1618*	29.9 (13.8) *n = 960*	28.9 (14.2) *n = 4545*
Swollen joint count (28)	20.9 (10.2) *n = 1618*	20.9 (9.8) *n = 960*	19.8 (9.9) *n = 4545*
HAQ-DI	1.5 (0.7) *n = 2559*	1.6 (0.7) *n = 1427*	1.6 (0.7) *n = 6895*
Patient pain (0–100 VAS)^†^	63.4 (21.0) *n = 2212*	63.4 (21.1) *n = 1173*	64.3 (21.1) *n = 5514*
**Concomitant medications**^**‡**^			
NSAIDs, *n* (%)	2238 (84.4)	1269 (85.5)	5668 (80.5)
Oral glucocorticoids, *n* (%)	1499 (56.5)	823 (55.4)	4285 (60.8)
Oral dose, mg	7.5 (8.1)	9.1 (9.1)	12.3 (162.5)
MTX, *n* (%)	1809 (68.2)	948 (63.8)	5184 (73.6)
Anti-TNF, *n* (%)	169 (6.4)	82 (5.5)	262 (3.7)

All values are mean (SD) unless otherwise stated

*HAQ-DI* health assessment questionnaire-disability index, *hsCRP* high-sensitivity C-reactive protein, *MTX* methotrexate, *NA* not available, *NSAID* non-steroidal anti-inflammatory drug, *TNF* tumor necrosis factor, *VAS* visual analog scale

*Excluding adalimumab arm in the AMPLE trial and infliximab arm in the ATTEST trial; background therapy, including methotrexate and corticosteroids, was permitted in the placebo arms

^†^VAS: 0 = no pain and 100 = worst possible pain

^‡^Up to the last dose of study medication

Tables 2 and 3 present the number and percentage of patients and IR (95% CI) for overall OI and the individual OIs reported in the controlled and cumulative periods.

**Table 2:** Incidence rates of OIs per 100 patient-years for OIs in controlled and cumulative periods

	Controlled periods				Cumulative periods	
Outcome	Abatacept: *n* = 2653; py = 2357		Placebo: *n* = 1485; py = 1254		Abatacept: *n* = 7044; py = 21,274	
	*n* (%)	IR/100 py (95% CI)	*n* (%)	IR/100 py (95% CI)	*n* (%)	IR/100 py (95% CI)
Opportunistic infections	4 (0.2)	0.17 (0.05–0.43)	7 (0.5)	0.56 (0.22–1.15)	45 (0.6)*	0.21 (0.15–0.28)*
Esophageal candidiasis	0	0	1 (0.1)	0.08 (0.0–0.4)	7 (0.1)	0.03 (0.01–0.07)
Pulmonary tuberculosis	0	0	0	0	6 (0.1)	0.03 (0.01–0.06)
Eye infection, fungal	1 (< 0.1)	0.04 (0.0–0.2)	0	0	3 (< 0.1)	0.01 (0–0.04)
Ophthalmic herpes simplex	0	0	0	0	3 (< 0.1)	0.01 (0–0.04)
Aspergillus infection	0	0	0	0	2 (< 0.1)	0.01 (0–0.03)
Blastocytosis infection	0	0	0	0	2 (< 0.1)	0.01 (0–0.03)
Bronchopulmonary aspergillosis	1 (< 0.1)	0.04 (0.0–0.2)	0	0	2 (< 0.1)	0.01 (0–0.03)
Lymph node tuberculosis	0	0	0	0	2 (< 0.1)	0.01 (0–0.03)
Respiratory moniliasis	0	0	1 (0.1)	0.08 (0.0–0.4)	2 (< 0.1)	0.01 (0–0.03)
Tuberculosis (unspecified)	1 (< 0.1)	0.04 (0–0.24)	1 (0.1)	0.08 (0–0.44)	2 (< 0.1)	0.01 (0–0.03)
Blastomycosis	0	0	0	0	1 (< 0.1)	0.00 (0–0.03)
Bone tuberculosis	0	0	0	0	1 (< 0.1)	0.00 (0–0.03)
Candida pneumonia	0	0	0	0	1 (< 0.1)	0.00 (0–0.03)
Cyclosporidium infection	0	0	0	0	1 (< 0.1)	0.00 (0–0.03)
Fungal esophagitis	0	0	1 (0.1)	0.08 (0.0–0.4)	1 (< 0.1)	0.00 (0–0.03)
Fungal pharyngitis	0	0	0	0	1 (< 0.1)	0.00 (0–0.03)
Cryptococcal meningitis	0	0	1 (0.1)	0.08 (0.0–0.44)	0	0
Gastrointestinal candidiasis	0	0	1 (0.1)	0.08 (0.0–0.44)	0	0
Gastrointestinal fungal infection	0	0	0	0	1 (< 0.1)	0.00 (0–0.03)
Histoplasmosis	0	0	0	0	1 (< 0.1)	0.00 (0–0.03)
Peritoneal tuberculosis	0	0	0	0	1 (< 0.1)	0.00 (0–0.03)
*Pneumocystis jirovecii* pneumonia	0	0	1 (0.1)	0.08 (0–0.4)	1 (< 0.1)	0.00 (0–0.03)
Pneumonia pseudomonal	1 (< 0.1)	0.04 (0.0–0.2)	0	0	1 (< 0.1)	0.00 (0–0.03)
Pseudomonas infection	0	0	0	0	1 (< 0.1)	0.00 (0–0.03)
Fungal sinusitis	0	0	0	0	1 (< 0.1)	0.00 (0–0.03)
Systemic candida	0	0	0	0	1 (< 0.1)	0.00 (0–0.03)
Systemic mycosis	0	0	0	0	1 (< 0.1)	0.00 (0–0.03)
Tuberculosis pleurisy	0	0	0	0	1 (< 0.1)	0.00 (0–0.03)
Pseudomonal urinary tract infection	0	0	0	0	1 (< 0.1)	0.00 (0–0.03)

*CI* confidence interval, *IR* incidence rate, *OI* opportunistic infection, *py* patient-years

*n (%) for SAE was 19 and IR/100 py was 0.1 (95% CI, 0.05–0.14)

**Table 3:** Incidence rates of tuberculosis and herpes per 100 patient-years in controlled and cumulative periods

	Controlled periods				Cumulative periods	
Outcome	Abatacept: *n* = 2653; py = 2357		Placebo: *n* = 1485; py = 1254		Abatacept: *n* = 7044; py = 21,274	
	*n* (%)	IR/100 py (95% CI)	*n* (%)	IR/100 py (95% CI)	*n* (%)	IR/100 py (95% CI)
Tuberculosis (overall)	1 (< 0.1)	0.04 (0–0.2)	1 (< 0.1)	0.08 (0–0.4)	13 (0.2)	0.06 (0.03–0.10)
Pulmonary tuberculosis	0	0	0	0	6 (0.1)	0.03 (0.01–0.06)
Lymph node tuberculosis	0	0	0	0	2 (< 0.1)	0.01 (0–0.03)
Tuberculosis (unspecified)	1 (< 0.1)	0.04 (0.0–0.2)	1 (0.1)	0.08 (0.0–0.4)	2 (< 0.1)	0.01 (0–0.03)
Bone tuberculosis	0	0	0	0	1 (< 0.1)	0.01 (0–0.03)
Peritoneal tuberculosis	0	0	0	0	1 (< 0.1)	0.01 (0–0.03)
Tuberculous pleurisy	0	0	0	0	1 (< 0.1)	0.01 (0–0.03)
Herpes simplex	57 (2.1)	2.5 (1.9–3.2)	22 (1.5)	1.8 (1.1–2.7)	60 (0.9)	1.48 (1.32–1.65)
Herpes zoster	44 (1.7)	1.9 (1.4–2.5)	21 (1.4)	1.7 (1.1–2.6)	284 (4.0)	1.53 (1.36–1.71)
Herpes virus infection (NOS)	5 (0.2)	0.2 (0.1–0.5)	4 (0.3)	0.3 (0.1–0.8)	20 (0.3)	0.09 (0.06–0.15)

*CI* confidence interval, *IR* incidence rate, *NOS* not otherwise specified, *py* patient-years

### Overall OIs

Overall OI IR (95% CI) for abatacept was similar and the corresponding 95% CIs were overlapping in both controlled (0.17 [0.05–0.43]) [17] and cumulative (0.21 [0.15–0.28]) periods. The overall OI IR (95% CI) for placebo in the controlled period was 0.56 (0.22–1.15).

Overall, 222 potential OI cases were evaluated and adjudicated. None of the patients had > 1 OI event in the controlled period; in the cumulative period, 3 patients had an instance of a recurrent OI (systemic candida, gastrointestinal fungal infection, esophageal candidiasis).

#### Controlled periods

In the controlled periods, the numbers of OIs reported for abatacept and placebo were 4 (0.2%) and 7 (0.5%) cases, respectively. Only single occurrences of OI events were reported. None resulted in death or discontinuation. The IR for overall OI for abatacept was lower compared with the IR for placebo (0.17 [0.05–0.43] versus 0.56 [0.22–1.15], respectively).

#### Cumulative period

In total, 45 (0.6%) subjects experienced OI events, with an IR (95% CI) of 0.21 (0.15–0.28). Of these, 19 subjects had events defined as SAEs, with an IR (95% CI) of 0.1 (0.05–0.14).

The most frequently reported OIs were esophageal candidiasis (*n* = 7 [0.1%], IR [95% CI] 0.03 [0.01–0.07]) and pulmonary TB (*n* = 6 [0.1%], IR [95% CI] 0.03 [0.01–0.06]).

### Tuberculosis

#### Controlled periods

Overall, there were 2 cases of TB reported (abatacept, *n* = 1; placebo, *n* = 1) with IRs (95% CIs) of 0.04 (0–0.2) for abatacept and 0.08 (0–0.4) for placebo.

#### Cumulative period

During the cumulative period, TB was reported in 13 (0.2%) abatacept-treated patients (IR [95% CI] 0.06 [0.03–0.10]). There were 6 cases of pulmonary TB; most of the patients (83%) were female with an age range of 39–64 years; geographically, cases were reported from Brazil (*n* = 1), Korea (*n* = 1), Mexico (*n* = 2), Portugal (*n* = 1), and Thailand (*n* = 1). One case of pulmonary TB resulted in death.

Five (31.3%) cases of TB were extrapulmonary (bone, lymph node, peritoneal, pleurisy). Most of the patients (60%) were female with an age range of 47–55 years; cases were reported from Argentina (*n* = 2), Brazil (*n* = 1), South Africa (*n* = 1), and Thailand (*n* = 1).

There were 2 cases of unspecified TB; both patients were female and aged 35 and 55 years (cases reported from Mexico [*n* = 1] and Peru [*n* = 1]).

Three (18.8%) cases of latent TB were reported and were not included in the overall TB IR; 2 (67%) patients were male; the patients were aged 43, 62, and 71 years, with cases reported from Mexico (*n* = 2) and Taiwan (*n* = 1). Cases of latent TB were not included in the IR of overall OIs.

### Herpes simplex virus and herpes zoster

Of the 284 herpes cases, 13 were reported as SAEs of herpes (9 zoster, 1 simplex, 1 dermatitis, 1 ophthalmic, and 1 virus infection). Herpes zoster was reported as a standard adverse event; therefore, additional clinical details were not collected. None of the herpes zoster cases were included in the overall OI IR.

#### Controlled periods

Herpes simplex and zoster were reported numerically more often with abatacept versus placebo in the controlled periods. There were 57 (2.1%) and 22 (1.5%) cases of herpes simplex and 44 (1.7%) and 21 (1.4%) cases of herpes zoster reported in the abatacept and placebo groups during the controlled periods. The IRs were similar and the corresponding 95% CIs were overlapping for herpes simplex (IR [95% CI] 2.5 [1.9–3.2] vs 1.8 [1.1–2.7]) and herpes zoster (IR [95% CI] 1.9 [1.4–2.5] vs 1.7 [1.1–2.6]), respectively.

#### Cumulative period

In the cumulative periods, the IRs (95% CI) for abatacept were lower for both herpes simplex (1.48 [1.32–1.65]) and herpes zoster (1.53 ([1.36–1.71]) compared with the IRs in the controlled periods. All AEs coded as herpes during the controlled periods were non-serious; 2 cases of herpes zoster infections (mild/grade I) with abatacept treatment, of which one was deemed probably related to treatment by the investigator, resulted in discontinuation of abatacept. During the cumulative periods, most of the AEs coded as herpes were herpes zoster and all were non-serious.

## Discussion

This analysis encompasses cumulative (controlled and open-label extension) OI data from 16 abatacept clinical trials with mean (SD) abatacept exposure of 36.9 (26.2) months and 21,274 total patient-years. Overall, the types of OIs reported in patients receiving abatacept were not unexpected. The rates of OIs between the abatacept and placebo groups were low and similar in the controlled periods. IRs of OIs over time remained stable with long-term abatacept use when considering open-label extension data. In the cumulative periods, the IR of OIs seen with abatacept was low and consistent with that observed in the controlled period.

The IR range of OIs observed for abatacept in this analysis (0.17–0.21 per 100 patient-years, across both periods) was generally consistent with the range reported in literature for other bDMARDs; in particular, TNF inhibitors (0.15–0.30 per 100 patient-years from a US study; notably, mycobacterial infections were included but shingles (herpes zoster) was excluded from the study by Baddley et al.) [20]. Similarly, another study from the British Society for Rheumatology Biologics Register for Rheumatoid Arthritis reported a crude IR for OIs (excluding tuberculosis) of 0.13 for all bDMARDs and TNF inhibitors, 0.15 for rituximab and 0.08 for tocilizumab [21]. In some published post-marketing observational studies, the rates (95% CI) of certain OIs for other bDMARDs varied considerably by treatment and geographic area: infliximab, 1.08 (0.37–3.22; Japan [22];), 8.0 (2.0–50.0; Spain [23]; infliximab versus etanercept, 17.6 (4.3–72.9), adalimumab versus etanercept, 10.28 (2.35–44.94; France [24]). There is some evidence to suggest a similar risk for OIs between some bDMARDs, but there may be differences for other OI outcomes (e.g., herpes zoster) [1]; hence, information is still lacking. Additionally, the IRs observed in this study are lower than those reported previously for JAK inhibitors. In an integrated analysis of 5671 patients treated with tofacitinib in phase 2 and 3 and long-term extension studies, the crude IR (95% CI) of OIs was 0.46 (0.36–0.59) per 100 patient-years [21]. In another recent pooled analysis of over 44 studies that included 48,093 patients exposed to a JAK inhibitor (tofacitinib, baricitinib, upadacitinib, or filgotinib), the IR of herpes zoster was 2.11 per 100 patient-years compared with 1.23 per 100 patient-years among patients exposed to a comparator [25].

Although patients receiving an active comparator (ATTEST: infliximab; AMPLE: adalimumab) were excluded from this analysis, previously published data from these studies indicate that OIs were less frequently reported with abatacept versus an active comparator. In the ATTEST study, no OIs were reported with abatacept, but two OIs occurred in the infliximab group by day 197 (controlled period, primary time point): a pseudomonal lung infection and a *Pneumocystis jiroveci* pneumonia [15]. The IR (95% CI) of OIs did not increase during abatacept treatment in the cumulative versus controlled period for patients who were treated with infliximab and switched to abatacept at day 365: 0.0 (0.0–0.0) versus 2.6 (0.7–6.6) [26]. In the AMPLE study, two cases of OI (1 with abatacept and 1 with adalimumab) occurred during the first year of treatment (controlled period); both were AEs of mucocutaneous oral candidiasis, and neither patient discontinued treatment [27]. In the 2-year cumulative period, 8 cases of OI occurred, 4 per treatment group: 1 case of histoplasmosis (AE) and 3 cases of oral candidiasis (1 SAE, 2 AE) with abatacept; 1 case of disseminated histoplasmosis (SAE), 2 cases of TB (miliary, pulmonary; both SAEs), and 1 oral candidiasis (AE) with adalimumab. None of the OIs in the abatacept group led to discontinuation, but both patients with reported TB in the adalimumab group discontinued the study [14].

The IR for TB observed in the cumulative data was low and similar to the IRs observed with abatacept versus placebo in the controlled periods of the clinical trials. The risk of TB in patients with RA has been shown to be elevated due to both the disease and the mechanism of action of many RA therapies, including steroids. However, risk of TB is largely driven by the country where such studies are conducted (i.e., endemic areas versus non-endemic areas) [28, 29]. While we observed a rate of 60/100,000 within the cumulative period, nearly all such cases occurred in countries with general population rates of TB between 20 and 80/100,000 person years. As such, one cannot exclude the possibility that some TB cases might be newly acquired in these endemic countries; reactivation of latent TB is also possible. In addition, although TB screening was applied at study entry, the possibility of some false negative results cannot be excluded. In a systematic literature review, seven observational studies addressing TB were identified; notably, most of them had a moderate or high risk of bias as per the Hayden’s tool [30]. The review showed an increased risk of TB in patients receiving TNF inhibitors, compared with the general population and with patients receiving csDMARDs (adjusted hazard ratio 2.7 to 12.5 per study) [30]. Whether abatacept substantially increases the risk of TB and its relative risk to other RA therapies, such as TNF antagonists, is unknown. To date, no such comparisons have been made in real-world data in areas endemic for TB. There have been limited direct comparisons in the context of randomized clinical trials, and in each of these small trials, fewer cases of TB were observed in patients treated with abatacept versus infliximab [15]. Regardless, given the potential risk of TB in patients with RA, it is important to screen for TB prior to starting any biologic therapy [31].

For the herpes outcomes, the cumulative IR was lower than the rate observed in the controlled period. Herpes zoster IRs reported in the literature for patients with RA who received biologic agents range from 1.61 to 2.71 per 100 patient-years [1, 3]. Notably, the data from both controlled (IR 1.9 per 100 patient-years) and cumulative (IR 1.53 per 100 patient-years) periods for herpes zoster lie within this reported range, supporting the currently known safety profile of abatacept. A systematic literature review used to inform the EULAR guidelines for the treatment of RA highlighted the lack of comparative data for risk of OIs with abatacept versus other bDMARDs and targeted synthetic (ts) DMARDs [32]; however, a recent study using data from 3 large US healthcare claims databases found that the risk of overall OIs and TB was similar with abatacept versus other b/tsDMARDs [33]. In the sensitivity analysis, an elevated risk of OIs for abatacept was observed in one of the three databases, which might be due to imbalances between the groups, such as greater co-medication differences in abatacept versus other b/tsDMARD initiators, or due to channeling bias [33].

In a previous analysis of tofacitinib in the real-world setting, the herpes zoster risk was significantly higher for tofacitinib versus abatacept, with adjusted hazard ratio of 2.01 (95% CI 1.40–2.88) [3].

There is inconsistency in how OIs are defined in the literature. Specifically, a full list of all OIs included in the definition is often omitted from publications. Thus, it is difficult to compare published OI rates unless a list of included OIs is provided by the authors. For this reason, defining OIs across clinical trial research programs can be problematic. Some efforts have been made to categorize OIs, including a consensus review and recommendations for reporting of OIs from clinical trials [2]. In the published review, the definition of an OI was formulated as “the presence, or specific presentation, of a pathogen that suggests a higher likelihood of an alteration in host immunity” [2]. In the present analysis, the authors applied this definition of an OI to the cases reported in these studies as a form of adjudication, to enhance clinical relevance and to improve generalizability of the findings. However, our adjudication efforts were limited as discussed below.

Certain strengths and limitations of this study should be considered when interpreting the results. Importantly, the analysis includes details not previously published on OI events reported in the cumulative periods of the trials, which allows for better generalizability of results, as the original controlled trial groups were maintained in the analysis [34]. The methodological approach of retrospectively adjudicating the OI cases resulted in difficulties in adjudicating some of the non-serious outcomes (herpes zoster) due to lack of clinical details collected at the time of the event. In this analysis, the IRs were computed for all reported infections as defined by the standard MedDRA terms, thus including the microorganisms and presentation that may not typically be considered as opportunistic (e.g., *Pseudomonas aeruginosa*-caused pneumonia; see Table 2) [2]; this limitation may have led to an overestimation of IRs. Lastly, each of the trials used in this analysis may be associated with general limitations common to all clinical trial studies. These include stringent patient eligibility criteria, which may lead to a specific patient population with fewer comorbidities but more severe RA at the start of the trial, compared with routine clinical practice. Some specific limitations of the clinical trials, such as sample size and short follow-up period (e.g., ACCOMPANY trial) [35, 36], should also be taken into account.

## Conclusions

In conclusion, this analysis of all randomized and long-term clinical trial data in patients with RA treated with abatacept (16 abatacept clinical trials representing over 7000 abatacept-treated patients, with 21,274 total patient-years) showed that abatacept-treated patients had a similar rate of OIs, compared with placebo-treated patients, in the controlled periods of the trials. The IRs of OIs were overall consistent in the cumulative periods for abatacept. No new or unexpected safety concerns were identified. The findings from this comprehensive analysis of IV and SC abatacept add valuable information on the safety profile of abatacept.

## Supplementary Information

**Additional file 1.**

## Data Availability

Bristol Myers Squibb policy on data sharing may be found at https://www.bms.com/researchers-and-partners/clinical-trials-and-research/disclosure-commitment.html.

## Abbreviations

AE: Adverse event
bDMARD: Biologic disease-modifying antirheumatic drug
CI: Confidence interval
csDMARD: Conventional synthetic disease-modifying antirheumatic drug
HAQ-DI: Health assessment questionnaire-disability index
hsCRP: High-sensitivity C-reactive protein
IR: Incidence rate
IV: Intravenous
MedDRA: Medical Dictionary for Regulatory Activities
MTX: Methotrexate
NA: Not available
NOS: Not otherwise specified
NSAID: Non-steroidal anti-inflammatory drug
OI: Opportunistic infection
RA: Rheumatoid arthritis
RCT: Randomized controlled trial
SAE: Serious adverse event
SC: Subcutaneous
SD: Standard deviation
TB: Tuberculosis
TNF: Tumor necrosis factor
tsDMARD: Targeted synthetic disease-modifying antirheumatic drug
VAS: Visual analog scale
