# Supplementary material for: Risk of opportunistic infections in patients with rheumatoid arthritis initiating abatacept: cumulative clinical trial data
Source: Arthritis Res Ther. 2021 Jan 11;23:17. doi: 10.1186/s13075-020-02399-2 (PMC7798209; doi:10.1186/s13075-020-02399-2)
Supplement: Supplementary file 1 — Additional file 1. [file 13075_2020_2399_MOESM1_ESM.docx]

**ADDITIONAL FILE 1**

**Supplementary Appendix A** Prespecified list of opportunistic infections in clinical trials

Abscess fungal

Actinomycotic sepsis

Acute pulmonary histoplasmosis

Adrenal gland tuberculosis

Allescheriosis

*Alternaria* infection

Arthritis fungal

Aspergilloma

Aspergillosis oral

*Aspergillus* infection

Atypical mycobacterial infection

Atypical mycobacterial lymphadenitis

Atypical mycobacterial pneumonia

Atypical mycobacterium pericarditis

*Aureobasidium pullulans* infection

BK virus infection

Biliary tract infection cryptosporidial

Biliary tract infection fungal

Bladder candidiasis

Blastocystis infection

Blastomycosis

Bone tuberculosis

Bovine tuberculosis

Bronchitis fungal

Bronchopulmonary aspergillosis

Bronchopulmonary aspergillosis allergic

*Burkholderia gladioli* infection

*Candida* endophthalmitis

*Candida* osteomyelitis

*Candida* pneumonia

*Candida* retinitis

*Candida* sepsis

Candidiasis of trachea

Capnocytophaga sepsis

Central nervous system fungal infection

Cerebral aspergillosis

Cerebral candidiasis

Cerebral fungal infection

Cerebral toxoplasmosis

Choroid tubercles

Chromoblastomycosis

Chronic pulmonary histoplasmosis

*Citrobacter* bacteremia

*Coccidioides* encephalitis

Coccidioidomycosis

Colitis herpes

Congenital tuberculosis

Conjunctivitis tuberculous

Cryptococcal cutaneous infection

Cryptococcal fungemia

Cryptococcosis

Cryptosporidiosis infection

Cutaneous blastomycosis

Cutaneous coccidioidomycosis

Cutaneous tuberculosis

*Cyclosporidium* infection

Cystitis pseudomonal

Cytomegalovirus chorioretinitis

Cytomegalovirus colitis

Cytomegalovirus duodenitis

Cytomegalovirus enteritis

Cytomegalovirus enterocolitis

Cytomegalovirus gastritis

Cytomegalovirus gastroenteritis

Cytomegalovirus gastrointestinal infection

Cytomegalovirus gastrointestinal ulcer

Cytomegalovirus hepatitis

Cytomegalovirus infection

Cytomegalovirus mucocutaneous ulcer

Cytomegalovirus myelomeningoradiculitis

Cytomegalovirus myocarditis

Cytomegalovirus nephritis

Cytomegalovirus esophagitis

Cytomegalovirus pancreatitis

Cytomegalovirus pericarditis

Cytomegalovirus syndrome

Cytomegalovirus urinary tract infection

Cytomegalovirus viremia

*Delftia acidovorans* infection

Disseminated Bacillus Calmette-Guerin infection

Disseminated cryptococcosis

Disseminated cytomegaloviral infection

Disseminated tuberculosis

Ear tuberculosis

Encephalitis cytomegalovirus

Encephalitis fungal

Encephalitis protozoal

Endocarditis candida

Endocarditis histoplasma

Endocarditis pseudomonal

Enterocolitis fungal

Epididymitis blastomyces

Epididymitis tuberculous

Erysipelothrix sepsis

Esophageal candidiasis

Esophageal tuberculosis

Exserohilum infection

Extrapulmonary tuberculosis

Eye infection fungal

Eye infection toxoplasmal

Female genital tract tuberculosis

Fungal abscess central nervous system

Fungal cystitis

Fungal endocarditis

Fungal labyrinthitis

Fungal esophagitis

Fungal peritonitis

Fungal pharyngitis

Fungal retinitis

Fungal tracheitis

Fungal urethritis

Fusarium infection

Gastritis fungal

Gastritis herpes

Gastroenteritis cryptococcal

Gastroenteritis cryptosporidial

Gastroenteritis pseudomonas

Gastrointestinal candidiasis

Gastrointestinal fungal infection

Geotrichum infection

Hemorrhagic pneumonia

Hepatic candidiasis

Hepatic infection fungal

Hepatitis toxoplasmal

Hepatosplenic candidiasis

Herpes esophagitis

Herpes sepsis

Herpes simplex colitis

Herpes simplex encephalitis

Herpes simplex gastritis

Herpes simplex hepatitis

Herpes simplex meningitis

Herpes simplex meningoencephalitis

Herpes simplex meningomyelitis

Herpes simplex necrotizing retinopathy

Herpes simplex esophagitis

Herpes simplex pneumonia

Herpes simplex sepsis

Herpes simplex viremia

Herpes simplex visceral

Herpes zoster cutaneous disseminated

Herpes zoster disseminated

Herpes zoster infection neurological

Herpes zoster meningitis

Herpes zoster meningoencephalitis

Herpes zoster meningomyelitis

Herpes zoster meningoradiculitis

Herpes zoster necrotizing retinopathy

Histoplasmosis

Histoplasmosis cutaneous

Histoplasmosis disseminated

Infective aneurysm

Intestinal tuberculosis

Isosporiasis

JC virus granule cell neuronopathy

Joint tuberculosis

Kaposi's varicelliform eruption

Listeremia

*Listeria* encephalitis

*Listeria* sepsis

Listeriosis

Lobomycosis

Lower respiratory tract herpes infection

Lower respiratory tract infection fungal

Lung infection pseudomonal

Lupus vulgaris

Lymph node tuberculosis

Lymphadenitis fungal

Male genital tract tuberculosis

Meningitis aspergillus

Meningitis candida

Meningitis coccidioides

Meningitis cryptococcal

Meningitis exserohilum

Meningitis fungal

Meningitis herpes

Meningitis histoplasma

Meningitis listeria

Meningitis toxoplasmal

Meningitis tuberculous

Meningoencephalitis herpes simplex neonatal

Meningoencephalitis herpetic

Meningomyelitis herpes

Microsporidia infection

Microsporum infection

Mucocutaneous candidiasis

Mucormycosis

Mycetoma mycotic

Mycobacterial infection

Mycobacterial peritonitis

Mycobacterium abscessus infection

Mycobacterium avium complex immune restoration disease

Mycobacterium avium complex infection

Mycobacterium chelonae infection

Mycobacterium fortuitum infection

Mycobacterium kansasii infection

Mycobacterium marinum infection

Mycobacterium ulcerans infection

Myocarditis mycotic

Myocarditis toxoplasmal

Necrotizing fasciitis fungal

Necrotizing herpetic retinopathy

*Neoscytalidium* infection

Neurocryptococcosis

Nocardia sepsis

Nocardiosis

Ophthalmic herpes simplex

Oral hairy leukoplakia

Oral tuberculosis

Oropharyngeal aspergillosis

Osseous cryptococcosis

Osteomyelitis blastomyces

Osteomyelitis fungal

Otitis media fungal

Pancreatitis fungal

*Paracoccidioides* infection

Parasitic pneumonia

Parvovirus B19 infection

Parvovirus infection

Penicillium infection

Pericarditis fungal

Pericarditis histoplasma

Pericarditis tuberculous

Peritoneal candidiasis

Peritoneal tuberculosis

Pheohyphomycosis

Pheohyphomycotic brain abscess

*Pneumocystis jirovecii* infection

*Pneumocystis jirovecii* pneumonia

Pneumonia blastomyces

Pneumonia cryptococcal

Pneumonia cytomegaloviral

Pneumonia fungal

Pneumonia herpes viral

Pneumonia pseudomonal

Pneumonia toxoplasmal

Presumed ocular histoplasmosis syndrome

Progressive multifocal leukoencephalopathy

Prostatitis tuberculous

Protothecosis

*Pseudallescheria* infection

*Pseudallescheria* sepsis

Pseudomonal bacteremia

Pseudomonal sepsis

*Pseudomonas aeruginosa* meningitis

*Pseudomonas* bronchitis

*Pseudomonas* infection

Pulmonary mycosis

Pulmonary paracoccidioidomycosis

Pulmonary sporotrichosis

Pulmonary trichosporonosis

Pulmonary tuberculoma

Pulmonary tuberculosis

Pyelonephritis fungal

Renal tuberculosis

Respiratory moniliasis

Respiratory tract infection fungal

Retinitis histoplasma

Rhinosporidiosis

Salpingitis tuberculous

*Scedosporium* infection

*Scopulariopsis* infection

Silicotuberculosis

Sinusitis aspergillus

Sinusitis fungal

*Sphingomonas paucimobilis* bacteremia

Spleen tuberculosis

Splenic candidiasis

Splenic infection fungal

Sporotrichosis

Superinfection fungal

Systemic candida

Systemic mycosis

Thyroid tuberculosis

*Torulopsis* infection

Toxoplasmosis

*Trichosporon* infection

Tuberculoma of central nervous system

Tuberculosis

Tuberculosis bladder

Tuberculosis gastrointestinal

Tuberculosis liver

Tuberculosis of central nervous system

Tuberculosis of eye

Tuberculosis of genitourinary system

Tuberculosis of intrathoracic lymph nodes

Tuberculosis of peripheral lymph nodes

Tuberculosis ureter

Tuberculous endometritis

Tuberculous laryngitis

Tuberculous pleurisy

Tuberculous tenosynovitis

Upper respiratory fungal infection

Urinary tract infection pseudomonal

Varicella zoster gastritis

Varicella zoster esophagitis

Varicella zoster pneumonia

Wound infection pseudomonas

*Yersinia* sepsi

**Supplementary Table 1** Clinical trials of abatacept in patients with RA included in the analysis

| Study | Patient population  (all: adults with active RA) | Intervention | Duration of short-term period (months) | Patients (*n*) | |
| --- | --- | --- | --- | --- | --- |
|  |  |  |  | Abatacept | Placebo |
| **AGREE** (NCT00122382) (1) | Early,* erosive, MTX-naïve | IV ABA or  PBO; +bg MTX | 12 | 256 | 253 |
| **AVERT** (NCT01142726) (1) | Early,* MTX- and biologic-naïve | SC ABA, SC ABA+MTX or PBO+MTX | 12 | ABA: 116; ABA+MTX: 119 | 116 |
| **ATTEST** (NCT00095147) (1)^†^ | Inadequate response to MTX | IV ABA, IFX or PBO; +bg MTX | 6 | 156 | 110 |
| **AIM** (NCT00048568) (1) | Inadequate response to MTX | IV ABA or PBO; +bg MTX | 12 | 433 | 219 |
| **IM101100** (NCT00162266) (1) | Inadequate response to MTX | IV ABA or PBO; +bg MTX | 12 | 220 | 119 |
| **IM101063** (NCT00254293) (1) | With bg DMARD | SC ABA or PBO; +bg DMARD | 3 | 51 | 17 |
| **ATTAIN** (NCT00048581) (1) | Inadequate response to MTX | IV ABA or PBO; +bg DMARDs | 6 | 258 | 133 |
| **ASSURE** (NCT00048932) (1) | With bg DMARDs and/or biologics | IV ABA or PBO; +bg RA therapy | 12 | 959 | 482 |
| **IM101101** (NCT00162279) (1) | Inadequate response to etanercept | IV ABA or PBO; +bg etanercept | 12 | 85 | 36 |
| **IM101015** (NCT00162201)(2) | Active RA, inadequate response to anti-TNF | IV ABA; +bg DMARDs | 4 | 16 | NA |
| **ARRIVE** (NCT00124982)(3) | Active RA,  inadequate response to anti-TNF | IV ABA; +bg DMARDs | 6 | Washout, *n* = 449; direct-switch, *n* = 597 | NA |
| **ACCOMPANY** (NCT00547521)(4, 5) | Active RA, inadequate response to ≥1 DMARD | SC ABA; +/-bg MTX | 4 | ABA + MTX, *n* = 51; ABA, *n* = 49 | NA |
| **ALLOW** (NCT00533897)(6) | Active RA, inadequate response to MTX | SC ABA; +bg MTX | 9 | 40 | 80^‡^ |
| **ACQUIRE** (NCT00559585)(7) | Active RA, inadequate response to MTX | IV or SC ABA;  +bg MTX | 6 | SC ABA, *n* = 736; IV ABA, *n* = 721 | NA |
| **ATTUNE** (NCT00663702)(8) | Active RA, completed ≥4 years in AIM or ATTAIN, inadequate response to MTX or anti-TNF | SC ABA | 12 | 123 | NA |
| **AMPLE** (NCT 00929864)(9) | Active RA, inadequate response to MTX, biologic-naïve | SC ABA vs SC adalimumab, both +bg MTX | 24 | 318 | NA |

*Early RA: disease duration ≤2 years, DAS28 (CRP) ≥3.2 at study entry; anti-CCP2 positive.

^†^Patients treated with infliximab were not included in this analysis (n = 165).

^‡^Period II

ABA=abatacept; ACCOMPANY=AbataCept in subjeCts with rheumatOid arthritis adMinistered Plus or minus background MTX subcutANeouslY; ACQUIRE=Abatacept Comparison of subQ versus intravenoUs in Inadequate Responders to methotrexatE; AGREE=Abatacept study to Gauge Remission and joint damage progression in methotrexate-naive patients with Early Erosive rheumatoid arthritis; AIM=Abatacept in Inadequate responders to MTX; ALLOW=Evaluation of Abatacept administered subcutaneousLy in aduLts with active rheumatOid arthritis: impact of Withdrawal and reintroduction on immunogenicity, efficacy and safety; AMPLE=Abatacept versus adaliMumab comParison in bioLogic-naïvE rheumatoid arthritis (RA) subjects with background methotrexate; ARRIVE=Abatacept Researched in Rheumatoid arthritis patients with an Inadequate anti-TNF response to Validate Effectiveness; ASSURE=Abatacept Study of Safety in Use with other RA therapies; ATTAIN=Abatacept Trial in Treatment of Anti-TNF INadequate responders; ATTEST=Abatacept or infliximab versus placebo, a Trial for Tolerability, Efficacy and Safety in Treating RA; ATTUNE=Abatacept in subjecTs who swiTch from intravenoUs to subcutaNeous thErapy; AVERT=Assessing Very Early Rheumatoid arthritis Treatment; bg=background; CCP2=cyclic citrullinated peptide-2; DAS28 (CRP)=Disease Activity Score 28 (C-reactive protein); DMARD=disease-modifying antirheumatic drug; IFX=infliximab; IV=intravenous; MTX=methotrexate; PBO=placebo; RA=rheumatoid arthritis; SC=subcutaneous.

**REFERENCES FOR SUPPLEMENTARY TABLE 1**

1. Simon T, Soule B, Hochberg M, Fleming D, Torbeyns A, Banerjee S, et al. Safety of abatacept versus placebo in rheumatoid arthritis: integrated data analysis of nine clinical trials. ACR Open Rheumatol. 2019;1(4):251-7.

2. Buch MH, Boyle DL, Rosengren S, Saleem B, Reece RJ, Rhodes LA, et al. Mode of action of abatacept in rheumatoid arthritis patients having failed tumour necrosis factor blockade: a histological, gene expression and dynamic magnetic resonance imaging pilot study. Ann Rheum Dis. 2009;68(7):1220-7.

3. Schiff M, Pritchard C, Huffstutter JE, Rodriguez-Valverde V, Durez P, Zhou X, et al. The 6-month safety and efficacy of abatacept in patients with rheumatoid arthritis who underwent a washout after anti-tumour necrosis factor therapy or were directly switched to abatacept: the ARRIVE trial. Ann Rheum Dis. 2009;68(11):1708-14.

4. Nash P, Nayiager S, Genovese M, Rodriguez C, Delaet I, Elegbe A, et al. Low immunogenicity, consistent safety and sustained clinical efficacy over 18 months of subcutaneous administration of abatacept with and without methotrexate in patients with rheumatoid arthritis-results from a phase III study. Ann Rheum Dis. 2010;69(Suppl III):iii97.

5. Nash P, Nayiager S, Genovese M, Kivitz A, Oelke K, Ludivico C, et al. Immunogenicity is not increased with subcutaneous administration of abatacept with and without methotrexate in patients with rheumatoid arthritis: results from a phase III study. Arthritis Rheum. 2009;60(10 Suppl):S633-4.

6. Kaine J, Gladstein G, Strusberg I, Robles M, Louw I, Gujrathi S, et al. Evaluation of abatacept administered subcutaneously in adults with active rheumatoid arthritis: impact of withdrawal and reintroduction on immunogenicity, efficacy and safety (phase IIIb ALLOW study). Ann Rheum Dis. 2012;71(1):38-44.

7. Genovese MC, Covarrubias A, Leon G, Mysler E, Keiserman M, Valente R, et al. Subcutaneous abatacept versus intravenous abatacept: a phase IIIb noninferiority study in patients with an inadequate response to methotrexate. Arthritis Rheumatol. 2011;63(10):2854-64.

8. Keystone EC, Kremer JM, Russell A, Box J, Abud-Mendoza C, Elizondo MG, et al. Abatacept in subjects who switch from intravenous to subcutaneous therapy: results from the phase IIIb ATTUNE study. Ann Rheum Dis. 2012;71(6):857-61.

9. Weinblatt ME, Schiff M, Valente R, van der Heijde D, Citera G, Zhao C, et al. Head-to-head comparison of subcutaneous abatacept versus adalimumab for rheumatoid arthritis: findings of a phase IIIb, multinational, prospective, randomized study. Arthritis Rheum. 2013;65(1):28-38.
